# Predicting Oral Drug Absorption: Mini Review on Physiologically-Based Pharmacokinetic Models

**DOI:** 10.3390/pharmaceutics9040041

**Published:** 2017-09-26

**Authors:** Louis Lin, Harvey Wong

**Affiliations:** Faculty of Pharmaceutical Sciences, University of British Columbia, Vancouver, BC V6T 1Z3, Canada; louis.lin@alumni.ubc.ca

**Keywords:** oral absorption, physiologically-based pharmacokinetic modeling, food-effect, pH effect, formulation simulation

## Abstract

Most marketed drugs are administered orally, despite the complex process of oral absorption that is difficult to predict. Oral bioavailability is dependent on the interplay between many processes that are dependent on both compound and physiological properties. Because of this complexity, computational oral physiologically-based pharmacokinetic (PBPK) models have emerged as a tool to integrate these factors in an attempt to mechanistically capture the process of oral absorption. These models use inputs from in vitro assays to predict the pharmacokinetic behavior of drugs in the human body. The most common oral PBPK models are compartmental approaches, in which the gastrointestinal tract is characterized as a series of compartments through which the drug transits. The focus of this review is on the development of oral absorption PBPK models, followed by a brief discussion of the major applications of oral PBPK models in the pharmaceutical industry.

## 1. Introduction

The oral route of drug administration is the most common, as opposed to injections (intravenous, intramuscular, subcutaneous) or inhalation, due to the convenience of administration. However, this route comes with the cost of absorption variability due to the many biological processes involved [1]. For example, variations in oral bioavailability can occur due to individual differences in the pre-systemic metabolism of drugs by the gut and liver. Two fundamental processes describing oral drug absorption include the dissolution of a drug into gastrointestinal (GI) fluid, and the permeation of a dissolved drug through the intestinal wall and into the bloodstream [2]. These processes are complex and are governed by physicochemical properties such as compound solubility and permeability. Other external physiological properties, including the pH environment and metabolic enzymes in the gastrointestinal tract (GIT), also play an important role in influencing oral absorption [3]. Furthermore, the complex interplay between these factors makes the prediction of the oral absorption of drugs difficult.

In the search for new medicines, low oral bioavailability is a common problem faced by scientists in the pharmaceutical industry [4,5]. Therefore, it is valuable to have methodologies that can assess the oral pharmacokinetics (PK) of drug candidates before moving forward with drug development. However, extensive in vivo evaluation in preclinical species can be costly and time consuming. Furthermore, species differences in pharmacokinetics can cause unreliable predictions of oral bioavailability in humans when using in vivo animal data [6,7]. In vitro assays assessing factors such as compound permeability, dissolution, solubility, and metabolic stability offer a more efficient and cost-effective means to evaluate the potential oral bioavailability of large numbers of drug candidates. However, these assays do not fully capture the complex and dynamic nature of the in vivo oral absorption process.

Mathematical models of oral absorption serve as tools to integrate compound data from in vitro assays gathered during the process of drug screening in order to provide in vivo context to these data. The focus of early mathematical models were almost solely on two fundamental compound properties, solubility and permeability. These earlier models were relatively simple and predicted the extent of absorption under more “static” conditions. Examples of these are the Absorption Potential (AP) and the Maximum Absorbable Dose (MAD) equations [8,9]. The AP equation is used to estimate the fraction absorbed of a given dose. The MAD equation estimates the maximum amount of a drug that can be absorbed within a 6-h time frame (meant to simulate small intestinal residence time), assuming drug saturation in the gastrointestinal fluid. Because of their simplicity, they enable rapid assessments of the possible extent of oral bioavailability from smaller information sets [10,11]. However, these models have limited ability to explore more complex phenomenon such as pH-dependent oral absorption and food effects.

**Figure 1:**
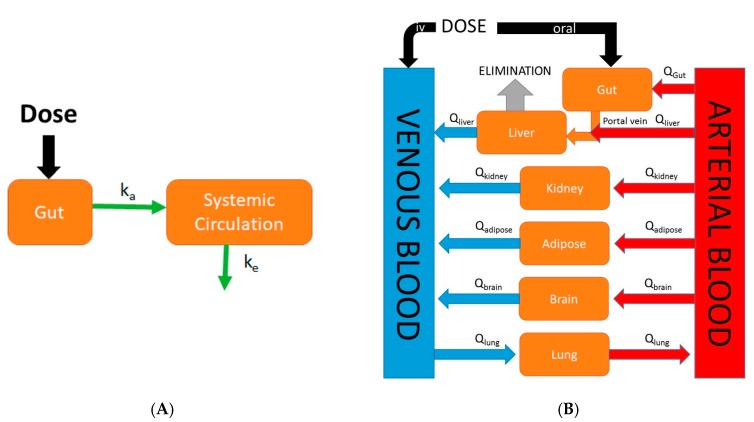
Comparison of an empirical classical compartmental model and a mechanistic physiologically-based pharmacokinetic (PBPK) model. (**A**) In the classical compartment model, a drug is inputted into the gut compartment, and absorption into the systemic circulation compartment is governed by the absorption rate constant (k_a_). Elimination is described by the elimination rate constant (k_e_); (**B**) In the whole-body PBPK model, major organs/tissues are represented by compartments, connected by blood flows (Q). Specific organ blood flows are described by subscripts. Intravenous (IV) dosing inputs drugs directly into venous blood, whereas oral dosing inputs drug into the gut compartment. In this illustration, the liver is the major eliminating organ.

Physiologically-based pharmacokinetic (PBPK) models are mathematical models that describe biological processes in order to mimic biology. They are dynamic in nature and are defined by series of differential equations. While classical compartmental pharmacokinetic models (Figure 1A) simply describe absorption as a single first-order process, PBPK models differ in that they are mechanistic in nature and incorporate physiological processes such as GI transit time and organ blood flows. Although the first PBPK model dates back to 1937, it was not until recently that the use of PBPK models in drug discovery research has rapidly increased in popularity [12]. This increase in popularity of PBPK modeling was largely due to the availability and speed at which ADME (absorption, distribution, metabolism, and excretion) information required as model inputs could be gathered due to advances in in silico and in vitro assays. An example of a generic whole-body PBPK model is shown in Figure 1B. Major tissues/organs are represented by compartments from which blood flows carry a drug into and out of tissue/organ. Specifically, in the case of oral PBPK models the gut is typically described as a series of compartments through which a drug transits (figures in Section 4 and Section 5). These models provide a rational means to translate preclinical absorption data to man. For example, one can validate a PBPK model of a particular drug built using rat in vitro data along with in vivo oral pharmacokinetic data in rat. Following the confirmation of a successful prediction of in vivo oral pharmacokinetics using in vitro data in the rat, the physiological parameters and in vitro parameters in the rat PBPK model can be replaced with human parameters in order to predict the human oral pharmacokinetic profile [13]. The use of PBPK modeling in pharmaceutical industry has rapidly expanded in recent times and has been used in sophisticated mechanistic applications such as the prediction of drug-drug interactions, the prediction of pharmacokinetic profiles in special populations, and the assessment of population variability. The specific focus of this review is on the evolution of physiologically-based pharmacokinetic models describing oral absorption. Further, we review the applications of these PBPK models of oral absorption in the current landscape of drug discovery and development.

## 2. Fundamental Processes for Oral Absorption

The fundamental processes that determine oral absorption include drug or compound dissolution and permeation. Dissolution is the process by which a solid drug dissolves into gastrointestinal fluid. Dissolution is normally described in PBPK models by a form of the Noyes-Whitney equation [14,15]:

(1) $$\frac{{dX}_{solution}}{dt}{=k}_{diss}\times \left(S-\frac{X_{solution}}{V}\right)$$

where *X_solution_* is the amount of drug in solution at time *t*, *V* is the volume of intestinal fluid, and *S* is the drug solubility. The rate at which the drug dissolves into solution follows first-order kinetics, dependent on the compound specific dissolution constant (*k_diss_*), and the concentration gradient surrounding the solid drug (*S* − [*X_solution_*/*V*]).

Following dissolution is permeation, the process by which a dissolved drug crosses the intestinal wall from the GI fluid into the portal vein. A drug is carried from the portal vein to the liver before reaching systemic circulation. Mathematically, permeation is described as a first-order differential equation governed by an absorption constant (k_a_) and the amount of drug in solution.

(2) $$\frac{{dX}_{perm}}{dt}{=k}_{a}{\times X}_{solution}$$

where *X_perm_* is the amount of permeated drug. The absorption rate constant (k_a_) can be calculated from effective permeability (*P_eff_*).

(3) $$k_{a}=\frac{{2P}_{eff}}{R}$$

where *R* refers to the radius of the small intestine. A compound’s *P_eff_* is calculated from the rate at which it permeates through a membrane (*dM_r_*/*dt*) using the following equation derived from Fick’s Law of Diffusion:

(4) $$P_{eff}=\frac{dM_{r}/dt}{A\times \left(C_{GIT}-C_{pv}\right)}$$

where *A* refers to the area of the membrane, *C_GIT_* refers to the concentration in the gastrointestinal tract, and *C_pv_* refers to the concentration that has permeated through the intestinal wall and into the portal vein. At the core of both dissolution and permeation equations is the principle of diffusion via a concentration gradient, reflected by (*C_GIT_* − *C_pv_*) for permeation and (*S* − [*X_solution_*/*V*]) for dissolution.

## 3. Mixing Tank Model

**Figure 2:**
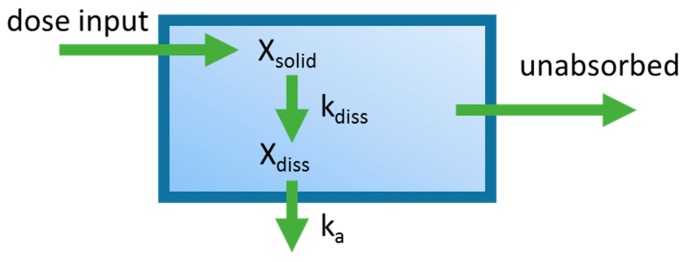
Diagram of the mixing tank model which represents the gastrointestinal (GI) tract as a single well-stirred compartment. k_a_ is the absorption rate constant, *X_diss_* is the amount of drug dissolved in the GI tract. *k_diss_* is the dissolution rate constant, and *X_solid_* is the dose that has been placed into the GI tract. The oral absorption rate is governed by k_a_ and *X_diss_*. The dissolution rate is governed by *X_solid_* and *k_diss_*.

One of the first applications integrating dissolution and permeation processes in an oral PBPK model was the mixing tank model, which treated the GIT as a single well-stirred compartment (Figure 2) [16]. The amount of drug inputted into the system was assumed to be instantaneously mixed throughout the GIT, and the movement of drug out of this single compartment was assumed to be governed by the intestinal transit time. This model was introduced by Dressman and Fleisher, and was one of the first models to allow for characterization of dissolution rate-limited absorption. Dressman et al. [16] validated this model’s ability to estimate the absorption profile for griseofulvin and digoxin using literature data. The model was then used to investigate factors limiting the oral absorption of these two compounds. Increasing dissolution rate and intestinal transit time using the model did not significantly increase bioavailability for griseofulvin, leading to the conclusion that drug solubility was the limiting factor. For digoxin, increasing the dissolution rate by decreasing particle size showed a significant increase in bioavailability, suggesting that the dissolution rate was limiting its absorption. A shortcoming of this early model is that it ignores phenomena such as gut metabolism, hepatic first-pass metabolism, and drug chemical instability. Furthermore, simplifying the entire GIT into a single homogenous compartment assumes that all dissolved drugs are subject to the same absorption rate constant, overlooking heterogeneity along the GIT. Despite the described shortcomings, the mixing tank model served to lay the foundations for the development of later oral PBPK models.

## 4. Compartmental Absorption and Transit Model

**Figure 3:**
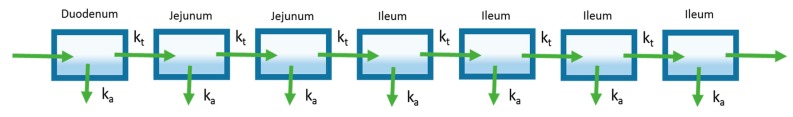
The compartmental absorption and transit (CAT) model extends the mixing tank model to characterize drug transit through the gastrointestinal tract (GIT). Seven well-stirred compartments are used to describe absorption and transit through the small intestine.

A later model of oral absorption that was introduced following the mixing tank model was the compartmental absorption and transit (CAT) model described by Yu et al. [17]. This model characterizes the intestinal tract as a series of compartments as opposed to a single compartment used by the mixing tank model. While a multiple compartment approach has been used prior to describe effects such as gastric emptying, Yu et al. utilized multiple compartments to represent different sections of the small intestine (Figure 3) [18,19,20,21]. Specifically, they found that the number of compartments that best fit the small intestine transit time, based on available literature, was seven. The first of the seven compartments represented the duodenum, the next two represented the jejunum, and the final four compartments represented the ileum. The transit of a drug through each small intestine compartment in CAT models is controlled by a transit rate constant (k_t_). The movement of drug through the CAT model can be mathematically represented by the following equation:

(5) $$\frac{{dY}_{n}}{dt}=k_{t}{\times Y}_{n-1}{-k}_{t}{\times Y}_{n}{-k}_{a}{\times Y}_{n}$$

where *Y_n_* refers to the amount of a drug in a specific compartment, *Y_n_*_−1_ refers to the amount of a drug in the previous compartment, k_t_ is the transit rate constant, and k_a_ is the absorption rate constant.

An advantage of the CAT model is its mathematic simplicity, as a single rate constant (k_t_) is used to describe the transit of a drug through different regions of the small intestine. Yu later added an additional seven compartments such that dissolved and undissolved drugs could be represented [22]. In this model, a rate constant describing dissolution governed the movement of a drug from undissolved compartments into the dissolved drug compartments. This addition allowed the CAT model to capture dissolution rate-limited absorption. The CAT model set the framework for future oral absorption models that incorporated additional features and properties to address its shortcomings.

## 5. Advanced Compartmental Absorption and Transit Model

**Figure 4:**
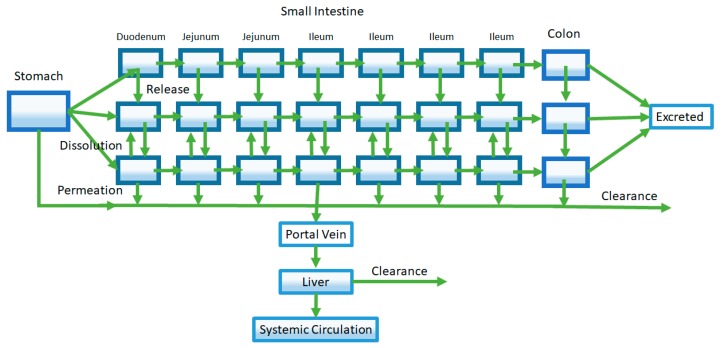
The representative Advanced Compartment Absorption and Transit (ACAT) model pictured here is an extension of the CAT model. Shown in this representative ACAT model, additional compartments are added to characterize features such as stomach and colon absorption, drug release from formulation, and first-pass metabolism from the liver and the gut (shown by the Clearance arrows).

Extensions of the early CAT model led to the development of the Advanced Compartment Absorption and Transit (ACAT) model that are implemented in the software GastroPlus^®^ [23]. An early form of the ACAT model adds new compartments describing three different drug states: unreleased drug in formulation, undissolved drug, and dissolved drug (Figure 4). As such, this ACAT model not only allows for investigation of dissolution-rate limited absorption, but also allows exploration of the effect of formulation release rates on oral pharmacokinetics. Like the CAT model, the small intestine is represented by seven compartments in series. In addition, the inclusion of stomach and colon segments allow for incorporation of processes such as gastric emptying and possible colonic absorption. Further, assignment of characteristics such as pH, effective surface area, transporter expression, and GI transit time to each specific compartment accounts for GI heterogeneity [24,25,26,27,28]. Finally, gut and liver metabolism were added to the ACAT model, improving predictions of the extent of oral absorption of drugs that undergo significant gut and liver first-pass metabolism such as propranolol [29].

Following the ACAT model, other similar advanced compartmental absorption models have been developed with slight differences in parameters and equations used. The most notable is the Advanced Dissolution Absorption Metabolism (ADAM) model incorporated in the SimCYP^®^ software, which uses the modified Wang and Flanagan method to model dissolution [30,31]. Among the PBPK models of oral absorption, the ACAT and ADAM models remain the most popular.

## 6. Incorporation of Saturable Processes: Metabolism and Drug Transporters

Metabolism and active transport are two important saturable processes that can have an influence on oral bioavailability [32,33]. As mentioned in the previous section, the incorporation of gut and liver metabolism in the ACAT model helped to correct for over-predictions of the extent of oral absorption for drugs that have significant gut and/or liver first-pass metabolism. A more complex situation is one where both metabolic enzymes and drug transporters are involved in oral absorption of a drug. Metabolic enzymes and drug transporters can have common substrates, which can lead to synergistic effects on oral absorption. Examples of this phenomenon include the impact of the efflux transporter P-glycoprotein and cytochrome P450 3A4 working synergistically to reduce the bioavailability of drugs such as cyclosporin and vinblastine [34,35,36,37].

The incorporation of saturable processes such as metabolism and drug transport into PBPK models are accomplished using Michaelis-Menten kinetics, which is described by the following equation:

(6) $$v=\frac{V_{max}\times [s]}{K_{m}+[s]}$$

where *v* refers to the rate the particular process being described, *V_max_* is the maximum rate of the process, K_m_ (Michaelis-Menten constant) is the concentration at which the rate is half maximal, and [*s*] is the compound (substrate) concentration. K_m_ can also be interpreted as the affinity for the compound to the enzyme or transporter. In cases where the drug concentrations are far below saturation, the equation can be shortened to:

(7) $$v=\frac{V_{max}\times [s]}{K_{m}}$$

**Figure 5:**
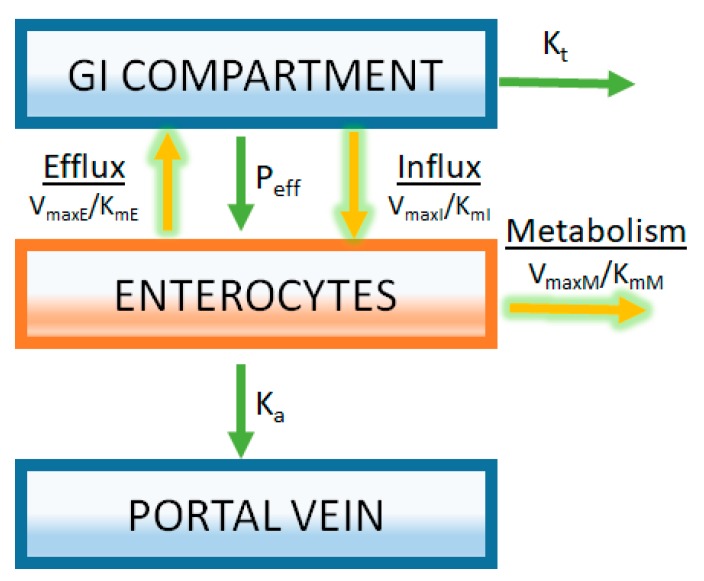
Modeling of transporters and intestinal metabolism is achieved by separately compartmentalizing the enterocytes. Rates of transport and metabolizing enzyme activity are described by Michaelis-Menten kinetics using parameters derived from in vitro enzyme activity assays (*V_max_*_E_, *V_max_*_I_, and *V_max_*_M_ are the maximum rate for efflux transporters, influx transporters, and metabolic enzymes respectively; K_mE_, K_mI_, and K_mM_ are the Michaelis-Menten constants for efflux transporters, influx transporters, and metabolic enzymes, respectively). In this example, it is assumed that substrate concentrations are far below saturation (Equation (7)).

For the case of liver metabolism, these kinetic parameters are commonly estimated in vitro by measuring enzyme activity using liver microsomes or hepatocytes [38,39,40]. A similar approach can be taken to estimate the kinetics of intestinal metabolism and transport using in vitro methodologies [41]. The success or failure of appropriately capturing the in vivo consequences of these processes on the oral absorption of drugs is highly dependent on the level of understanding how in vitro data generated for these processes translate to an in vivo system. While there is much work performed to understand the in vitro to in vivo translation of metabolic enzyme activity obtained from in vitro experiments, less is known in this regard for drug transport. An example of how saturable processes are incorporated into an oral PBPK model is illustrated in Figure 5. A gut segment is represented in the figure as a GI compartment (representing the lumen) with a separate enterocyte compartment. A drug must pass through the enterocyte compartment prior to being absorbed into the portal vein. Michaelis-Menten kinetics is used to represent both the metabolism and transport processes (Figure 5). Having separate gut segments representing the gastrointestinal tract allows ACAT models to capture the heterogeneity of expression of transporter and enzymes.

## 7. Applications of Oral PBPK Models

PBPK models can have many different applications during the course of drug discovery and development. Simulations using oral PBPK models built using limited drug discovery data can be used to assess the oral absorption of drug candidates by providing in vivo context to all available in vitro data. These early PBPK models have been used to assess oral pharmacokinetics of drug candidates in humans. More detailed simulations can be performed using more refined oral PBPK models at later stages of drug development to inform decisions about drug form selection and formulation optimization. Further, oral PBPK models offer an ideal tool to explore complex dynamic phenomenon such as pH and food effects. The applications of oral PBPK models to guide drug form selection and formulation optimization as well as to investigate pH and food effects are of high value. A more detailed look at these applications is described below.

### 7.1. Drug Form Selection and Formulation Optimization

Of the various applications of oral PBPK models, one of the applications that has great value is in the area of drug form selection and formulation optimization. The oral absorption of drugs is highly dependent on various drug specific properties such as particle size and drug solubility of different drug forms. Oral PBPK models can be utilized to quantify how changes in these properties influence oral absorption through the use of sensitivity analysis. Sensitivity analysis involves the systematic alteration of a specific drug parameter (e.g., particle size) in an oral PBPK model in order to identify the optimal values required for maximizing oral absorption. Below are descriptions of two studies where sensitivity analysis was used for salt selection and particle size optimization.

#### 7.1.1. Salt Selection

Salt selection for new molecular entities remains a challenge, due to the difficulties in translating in vitro solubility to in vivo bioavailability. For most cases, salt forms with the highest solubility are selected with little regard for other factors (i.e., which salt form has the best solid-state properties for further drug development). In order to assess salt forms with an in vivo context, Chiang and Wong used an oral PBPK model to explore the salt solubility-bioavailability relationship for phenytoin [42]. The exercise identified a solubility of ~0.3 mg/mL that was required in order to produce the maximum bioavailability of phenytoin. Any increase in solubility beyond 0.3 mg/mL was predicted to provide no additional increases in oral bioavailability, as drug solubility would no longer be rate limiting for oral absorption. Phenytoin salts with a wide range of solubility were prepared and administered orally to rats. Despite having a 60-fold difference in solubility, two phenytoin salts—both with solubility >0.3 mg/mL—provided a similar oral bioavailability as predicted by the oral PBPK analysis. This study demonstrates that oral PBPK models can be used to define solubility requirements and provide guidance for salt selection.

#### 7.1.2. Particle Size

A critical factor influencing the oral absorption of poorly soluble drugs is the drug dissolution rate that is dependent on the drug particle size. Particle size of drug product is a characteristic that can be controlled. Therefore, the identification of an optimal particle size is an important consideration in drug development, especially for compounds showing dissolution-rate limited oral absorption. In the Noyes-Whitney equation for dissolution (Equation (1)), particle size is incorporated in the calculation for the dissolution constant.

(8) $$k_{\mathrm{diss}}=\frac{{3\times D}_{eff}\times S}{r_{p}^{2}\times \rho }$$

where k_diss_ is the dissolution constant, *D_eff_* is the effective diffusion coefficient, *ρ* is the drug density, *r_p_* is the particle size, and *S* is the solubility. Based on the Noyes-Whitney equation, a smaller particle size (i.e., smaller *r_p_*) translates to more rapid dissolution, due to the increases in the drug particle surface area. However, smaller particle size can require extensive milling of drug products and additional workup.

An example of using PBPK modeling to understand the effect of compound particle size on its in vivo dissolution was reported by Parrott and Lave [43]. As a first step of PBPK model verification, the particle size distribution of a milled and an un-milled compound was characterized. Next, the milled and un-milled compounds were formulated into tablets and administered to monkeys. Particle size distribution data was used as input into a monkey PBPK model, and simulations of the oral monkey plasma concentration-time profiles for the milled and un-milled compounds were performed. PBPK model simulations were compared to in vivo plasma-concentration time profiles from monkey as a means to verify the PBPK model’s ability to predict the in vivo dissolution of the milled and un-milled compounds using particle size data. Following model verification, a human PBPK model was used to perform a sensitivity analysis examining the effect of particle size on maximum concentration (C_max_) and total drug exposure (AUC). The sensitivity analysis suggested that C_max_ was more sensitive to particle changes when compared to AUC. A particle diameter above 24 µm would decrease C_max_ by 20%, whereas a particle diameter above 44 μm would be required for a 20% decrease in AUC. The information from this analysis exemplifies how oral PBPK models can be used to provide guidance on optimal particle size ranges to produce the desired in vivo dissolution profile and resultant oral pharmacokinetic profile.

### 7.2. Food and pH Effects

A second area of application in which oral PBPK models can serve as a useful tool is in the investigation of pH and food effects on oral bioavailability [44,45,46]. Of the two phenomena, pH effects are much easier to deal with using PBPK models. As the gastrointestinal tract is represented as a series of compartments in oral PBPK models, a pH effect can be simulated by simply adjusting the pH in the GI compartment of interest. The alteration of pH primarily affects the solubility of the solid drug in the specific compartment, and subsequent simulation with the PBPK model will indicate its effect on oral bioavailability. A more complex phenomenon is food effects on oral absorption. Following food intake, changes in GI transit and gastric pH occur [47]. Further, bile secretions from the gall bladder aid in the dissolution and permeation of lipophilic compounds [48]. Due to the many physiological changes that occur with food intake and the potential for direct food-drug interaction, PBPK modeling of food effects is more challenging. However, as GI physiology can differ between animals and humans, human PBPK models combined with in vitro data may be the most appropriate means to capture and predict how drugs are absorbed in the human fasted and fed states.

#### 7.2.1. Investigating pH Effect

An oral PBPK model was used to investigate the cause of an observed disproportionate increase in drug exposure with increasing dose for ARRY-403 in a single ascending dose study [46]. Findings from the analysis suggested that ARRY-403 dissolved completely in the stomach. The disproportionate increase in ARRY-403 exposure with dose was attributed to dose-limited absorption resulting from ARRY-403’s marked pH-dependent solubility. As ARRY-403 had a marked pH-dependent solubility, simulations were performed using the PBPK model to examine the effect of acid-reducing agents on its pharmacokinetics. The PBPK simulations suggested that a clinical study with an acid-reducing agent was warranted. An ARRY-403 clinical study with famotidine co-administration confirmed an acid-reducing agent effect on ARRY-403 exposure that was predicted from model simulations. This example demonstrates the utility of PBPK models for investigation of pH effects on oral absorption.

#### 7.2.2. Investigating Mechanisms of Food Effect

An example of a food effect investigation using PBPK modeling is found in a reported investigation of a decrease in zolpidem drug exposure following administration of a modified release formulation with food [49]. Investigation of the mechanisms of this “negative food effect” was performed using biorelevant dissolution testing and commercially available PBPK modeling software. Based on the analysis, it appeared that the absorption of zolpidem is largely determined by the gastric emptying of the formulation. Because of this, considerable intra- and inter-subject variability in the onset of drug absorption was observed, especially for the immediate release formulation. Further, the release of the zolpidem appeared to be impaired by a high-fat and/or protein environment for both the immediate and modified release formulation.

A second example of using PBPK models to explore the mechanism of an observed food effect was an exploration of a positive food effect of compound X [50]. Compound X is a weak base with pH-dependent solubility that exhibited a significant dose-dependent food effect in humans. As part of the PBPK analysis, a parameter sensitivity analysis was performed to evaluate the effect of particle size on the oral absorption of compound X. It was shown that the oral absorption of compound X can be increased by reducing the particle size (<100 nm) of the active pharmaceutical ingredient under fasted conditions. Therefore, it follows that a reduction of particle size in the formulation of compound X would serve as a mitigation strategy to reduce its food effect.

The two studies described above illustrate the how PBPK models are used in investigations of food effect in humans. Further they illustrate the integration of PBPK models as important tools that can provide an understanding of the mechanisms responsible for observed food effects.

### 7.3. Predicting Human Oral Pharmacokinetics

A third application of PBPK models of oral absorption includes early attempts to predict human pharmacokinetics following oral dosing of drug candidates. These predictions often occur during the drug discovery phase, and with limited PBPK model verification in humans. An example of this application was described by Liu et al., where the oral pharmacokinetic properties in humans of drug candidate YQA-14, a dopamine D_3_ receptor antagonist, was successfully predicted [51]. The authors first verified the PBPK model by comparing the performance of simulations using rat and dog PBPK models with preclinical pharmacokinetic data from rats and dogs, respectively. PBPK models of YQA-14 in rat and dog used in vitro microsomal stability data as model inputs. Both models predicted in vivo PK of the respective species within two-fold of the observed values. Following PBPK model verification, a human PBPK model was built again using in vitro microsomal stability data. This human PBPK model was to simulate the PK behavior of YQA-14 in humans. The resulting human PK simulations were deemed acceptable for further development of the compound. While this study demonstrates some value in using oral PBPK models to predict human oral pharmacokinetics in drug discovery, it does not speak to the performance of such models.

A recent PhRMA (Pharmaceutical Research and Manufacturers of America) initiative assessed the quality of PBPK models in predicting oral human PK profiles of 108 compounds using preclinical data in a blinded manner. The study found that only 23% of oral drug simulations had a medium-high accuracy (up to two-fold error) [52]. Simulations had a general underestimation of oral absorption. Furthermore, inaccuracies mostly occurred in drugs with poorly soluble characteristics, which presents a bigger problem in the context of the current trend of increasing lipophilicity in new chemical entities (NCE) [53]. This study points to the idea that PBPK models using preclinical data perform poorly in predicting oral human pharmacokinetic profiles and highlights a need to continue improving our understanding of oral absorption.

### 7.4. Performance of Various Applications of PBPK Models of Oral Absorption

Of the three areas of application summarized (Section 7.1, Section 7.2 and Section 7.3), evidence in the literature of the poor performance of PBPK models of oral absorption appears largely for the prediction of human oral pharmacokinetics. Typically, the prediction of human oral pharmacokinetics occurs at the transition stage between late drug discovery and early drug development. Less ADME and physicochemical information is available for drug candidate compounds at this transition stage, making it more difficult to build a PBPK model with acceptable performance. Further, human oral PK predictions are made prior to having in vivo human pharmacokinetic data available. In contrast, oral PBPK models used to examine drug form selection and formulation optimization often have the benefit of being more refined. These PBPK models may have been verified using in vivo human PK data available from Phase 1 trials, as much of this work is performed following the initial Phase 1 trial. The same can be said for oral PBPK models used to investigate food and pH effects. The availability of more mechanistic information at later stages of drug development likely provides performance advantages to those PBPK models used to investigate drug form selection, formulation optimization, and food and pH effects when compared to the earlier PBPK models used to predict human oral PK. A challenge will be to improve the performance of PBPK models at earlier stages of drug development where less compound-specific information is available. A better understanding of the in vivo relevance of preclinical data used to build PBPK models may serve to improve predictions of human oral pharmacokinetics of new drug candidates in the long term.

### 7.5. Model Verification/Validation

Successful application of PBPK models of oral absorption require iterative cycles of model verification/validation involving a comparison of observed pharmacokinetic data to that from PBPK model simulations. The poor performance of the “blinded” PBPK predictions of oral human PK of 108 compounds performed by the PhRMA initiative [51] speaks loudly to the importance of verification and re-verification of PBPK models as new data becomes available.

**Figure 6:**
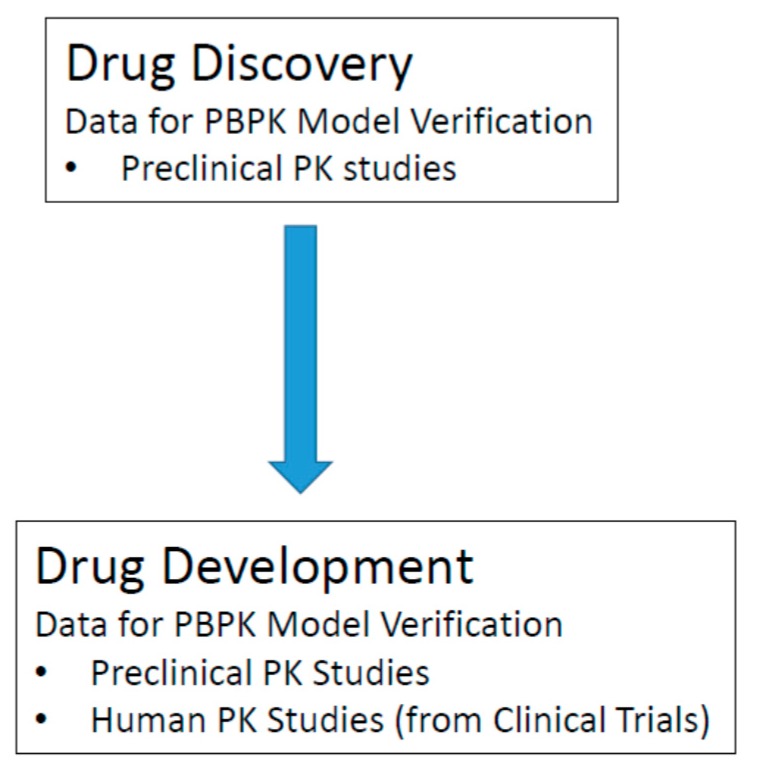
Sources of PK data required for model verification.

In the drug discovery phase, only preclinical PK data is available for model verification (Figure 6). At this stage, PBPK model verification consists of a comparison between in vivo PK data typically from rat, dog, or monkey to simulations from PBPK models of rat, dog, or monkey, respectively. At this early phase, the importance of PBPK model verification is to determine if you have identified the important preclinical data inputs (usually in vitro data) to enable your PBPK model to predict the in vivo drug disposition of your compound of interest. If successful predictions are made in preclinical species, human oral PK predictions can be performed with a higher level of confidence. In the drug development phase, both preclinical and human PK data is available for model verification. The availability of human data at this later phase plays a big role in improving the performance of PBPK models developed during clinical development. An advantage of initiating PBPK model-building earlier during drug discovery is that it provides an opportunity for more iterations of model verification and refinement. In a sense, an evolving PBPK model that is initially built during drug discovery can serve as a means to capture in vivo relevant ADME information for compounds of interest. This evolving PBPK model can be passed on to new scientists working on a particular compound as it progresses forward in the drug development process.

## 8. Conclusions

Computational oral absorption models, in particular PBPK models, provide a powerful tool for researchers and pharmaceutical scientists in drug discovery and development, as they mimic physiologically processes relevant to oral absorption. PBPK models provide in vivo context to in vitro data and allow for a dynamic understanding of in vivo drug disposition that is not typically provided by data from standard in vitro assays. Investigations using oral PBPK models enable informed decision-making, especially with respect to formulation strategies in drug development. PBPK models can also be used to investigate and provide insight into mechanisms responsible for complex phenomena such as food effects.

Ongoing research in the area of oral absorption will increase understanding and allow for the refinement of oral PBPK models. As our understanding of oral absorption improves, the ability of PBPK models to predict oral pharmacokinetics will also improve, providing a better tool for the discovery and development of new medicines in the pharmaceutical industry.

## References

[1] Sugihara M., Takeuchi S., Sugita M., Higaki K., Kataoka M., Yamashita S. (2015). Analysis of intra- and intersubject variability in oral drug absorption in human bioequivalence studies of 113 generic products. Mol. Pharm..

[2] Martinez M.N., Amidon G.L. (2002). A mechanistic approach to understanding the factors affecting drug absorption: A review of fundamentals. J. Clin. Pharmacol..

[3] Mudie D.M., Amidon G.L., Amidon G.E. (2010). Physiological parameters for oral delivery and in vitro testing. Mol. Pharm..

[4] Waring M.J., Arrowsmith J., Leach A.R., Leeson P.D., Mandrell S., Owen R.M., Pairaudeau G., Pennie W.D., Pickett S.D., Wang J. (2015). An analysis of the attrition of drug candidates from four major pharmaceutical companies. Nat. Rev. Drug Discov..

[5] Ward K.W. (2012). Optimizing pharmacokinetic properties and attaining candidate selection. Reducing Drug Attrition.

[6] Chu X., Bleasby K., Evers R. (2013). Species differences in drug transporters and implications for translating preclinical findings to humans. Expert Opin. Drug Metab. Toxicol..

[7] Chanteux H., Staelens L., Mancel V., Gerin B., Boucaut D., Prakash C., Nicolas J.-M. (2015). Cross-species differences in the preclinical pharmacokinetics of CT7758, an α4β1/α4β7 integrin antagonist. Drug Metab. Dispos. Biol. Fate Chem..

[8] Dressman J.B., Amidon G.L., Fleisher D. (1985). Absorption potential: Estimating the fraction absorbed for orally administered compounds. J. Pharm. Sci..

[9] Johnson K.C., Swindell A.C. (1996). Guidance in the setting of drug particle size specifications to minimize variability in absorption. Pharm. Res..

[10] Ding X., Rose J.P., Van Gelder J. (2012). Developability assessment of clinical drug products with maximum absorbable doses. Int. J. Pharm..

[11] Sun D., Yu L.X., Hussain M.A., Wall D.A., Smith R.L., Amidon G.L. (2004). In vitro testing of drug absorption for drug “developability” assessment: Forming an interface between in vitro preclinical data and clinical outcome. Curr. Opin. Drug Discov. Devel..

[12] Torsten T. (1937). Kinetics of distribution of substances administered to the body, I: The extravascular modes of administration. Arch. Int. Pharmacodyn. Ther..

[13] Jones H.M., Parrott N., Jorga K., Lavé T. (2006). A Novel strategy for physiologically based predictions of human pharmacokinetics. Clin. Pharmacokinet..

[14] Noyes A.A., Whitney W.R. (1897). The rate of solution of solid substances in their own solutions. J. Am. Chem. Soc..

[15] Sugano K., Okazaki A., Sugimoto S., Tavornvipas S., Omura A., Mano T. (2007). Solubility and dissolution profile assessment in drug discovery. Drug Metab. Pharmacokinet..

[16] Dressman J.B., Fleisher D. (1986). Mixing-tank model for predicting dissolution rate control or oral absorption. J. Pharm. Sci..

[17] Yu L.X., Crison J.R., Amidon G.L. (1996). Compartmental transit and dispersion model analysis of small intestinal transit flow in humans. Int. J. Pharm..

[18] Dressman J.B., Fleisher D., Amidon G.L. (1984). Physicochemical model for dose-dependent drugabsorption. J. Pharm. Sci..

[19] Oberle R.L., Amidon G.L. (1987). The influence of variable gastric emptying and intestinal transit rates on the plasma level curve of cimetidine; an explanation for the double peak phenomenon. J. Pharmacokinet. Biopharm..

[20] Luner P.E., Amidon G.L. (1993). Description and simulation of a multiple mixing tank model to predict the effect of bile sequestrants on bile salt excretion. J. Pharm. Sci..

[21] Bailey J.M., Shafer S.L. (1991). A simple analytical solution to the three-compartment pharmacokinetic model suitable for computer-controlled infusion pumps. IEEE Trans. Biomed. Eng..

[22] Yu L.X. (1999). An integrated model for determining causes of poor oral drug absorption. Pharm. Res..

[23] Agoram B., Woltosz W.S., Bolger M.B. (2001). Predicting the impact of physiological and biochemical processes on oral drug bioavailability. Adv. Drug Deliv. Rev..

[24] Jambhekar S.S., Breen P.J. (2013). Drug dissolution: Significance of physicochemical properties and physiological conditions. Drug Discov. Today.

[25] Dressman J.B., Vertzoni M., Goumas K., Reppas C. (2007). Estimating drug solubility in the gastrointestinal tract. Adv. Drug Deliv. Rev..

[26] Mouly S., Paine M.F. (2003). P-glycoprotein increases from proximal to distal regions of human small intestine. Pharm. Res..

[27] Ungell A.L., Nylander S., Bergstrand S., Sjöberg A., Lennernäs H. (1998). Membrane transport of drugs in different regions of the intestinal tract of the rat. J. Pharm. Sci..

[28] Paine M.F., Khalighi M., Fisher J.M., Shen D.D., Kunze K.L., Marsh C.L., Perkins J.D., Thummel K.E. (1997). Characterization of interintestinal and intraintestinal variations in human CYP3A-dependent metabolism. J. Pharmacol. Exp. Ther..

[29] Sawamoto T., Haruta S., Kurosaki Y., Higaki K., Kimura T. (1997). Prediction of the plasma concentration profiles of orally administered drugs in rats on the basis of gastrointestinal transit kinetics and absorbability. J. Pharm. Pharmacol..

[30] Jamei M.Y.J. A Novel Physiologically-Based Mechanistic Model for Predicting Oral Drug Absorption: The Advanced Dissolution, Absorption, and Metabolism (ADAM) Model. https://www.escholar.manchester.ac.uk/uk-ac-man-scw:108992.

[31] Wang J., Flanagan D.R. (1999). General solution for diffusion-controlled dissolution of spherical particles. 1. Theory. J. Pharm. Sci..

[32] Murakami T., Takano M. (2008). Intestinal efflux transporters and drug absorption. Expert Opin. Drug Metab. Toxicol..

[33] Hurst S., Loi C.-M., Brodfuehrer J., El-Kattan A. (2007). Impact of physiological, physicochemical and biopharmaceutical factors in absorption and metabolism mechanisms on the drug oral bioavailability of rats and humans. Expert Opin. Drug Metab. Toxicol..

[34] Chan L.M.S., Cooper A.E., Dudley A.L.J., Ford D., Hirst B.H. (2004). P-glycoprotein potentiates CYP3A4-mediated drug disappearance during Caco-2 intestinal secretory detoxification. J. Drug Target..

[35] Wacher V.J., Silverman J.A., Zhang Y., Benet L.Z. (1998). Role of P-glycoprotein and cytochrome P450 3A in limiting oral absorption of peptides and peptidomimetics. J. Pharm. Sci..

[36] Lown K.S., Mayo R.R., Leichtman A.B., Hsiao H.L., Turgeon D.K., Schmiedlin-Ren P., Brown M.B., Guo W., Rossi S.J., Benet L.Z. (1997). Role of intestinal P-glycoprotein (MDR1) in interpatient variation in the oral bioavailability of cyclosporine. Clin. Pharmacol. Ther..

[37] Wacher V.J., Wu C.Y., Benet L.Z. (1995). Overlapping substrate specificities and tissue distribution of cytochrome P450 3A and P-glycoprotein: Implications for drug delivery and activity in cancer chemotherapy. Mol. Carcinog..

[38] Kumar S., Samuel K., Subramanian R., Braun M.P., Stearns R.A., Chiu S.-H.L., Evans D.C., Baillie T.A. (2002). Extrapolation of diclofenac clearance from in vitro microsomal metabolism data: Role of Acyl glucuronidation and sequential oxidative metabolism of the Acyl glucuronide. J. Pharmacol. Exp. Ther..

[39] Kusuhara H., Sugiyama Y. (2009). In vitro-in vivo extrapolation of transporter-mediated clearance in the liver and kidney. Drug Metab. Pharmacokinet..

[40] Kitamura S., Maeda K., Sugiyama Y. (2008). Recent progresses in the experimental methods and evaluation strategies of transporter functions for the prediction of the pharmacokinetics in humans. Naunyn-Schmiedebergs Arch. Pharmacol..

[41] Gertz M., Houston J.B., Galetin A. (2011). Physiologically based pharmacokinetic modeling of intestinal first-pass metabolism of CYP3A substrates with high intestinal extraction. Drug Metab. Dispos..

[42] Chiang P.-C., Wong H. (2013). Incorporation of physiologically based pharmacokinetic modeling in the evaluation of solubility requirements for the salt selection process: A case study using phenytoin. AAPS J..

[43] Parrott N., Lave T. (2008). Applications of physiologically based absorption models in drug discovery and development. Mol. Pharm..

[44] Abuhelwa A.Y., Williams D.B., Upton R.N., Foster D.J.R. (2017). Food, gastrointestinal pH, and models of oral drug absorption. Eur. J. Pharm. Biopharm..

[45] Li X., Shi L., Tang X., Wang Q., Zhou L., Song W., Feng Z., Ge J., Li J.K., Yang L. (2017). Mechanistic prediction of food effects for Compound A tablet using PBPK model. Saudi J. Biol. Sci..

[46] Chung J., Alvarez-Nunez F., Chow V., Daurio D., Davis J., Dodds M., Emery M., Litwiler K., Paccaly A., Peng J. (2015). Utilizing physiologically based pharmacokinetic modeling to inform formulation and clinical development for a Compound with pH-dependent solubility. J. Pharm. Sci..

[47] Welling P.G. (1996). Effects of food on drug absorption. Annu. Rev. Nutr..

[48] Glomme A., März J., Dressman J.B., Testa B., Krämer S.D., Wunderli-Allenspach H., Folkers G. (2006). Predicting the intestinal solubility of poorly soluble drugs. Pharmacokinetic Profiling in Drug Research.

[49] Andreas C.J., Pepin X., Markopoulos C., Vertzoni M., Reppas C., Dressman J.B. (2017). Mechanistic investigation of the negative food effect of modified release zolpidem. Eur. J. Pharm. Sci..

[50] Zhang H., Xia B., Sheng J., Heimbach T., Lin T.-H., He H., Wang Y., Novick S., Comfort A. (2014). Application of physiologically based absorption modeling to formulation development of a low solubility, low permeability weak base: Mechanistic investigation of food effect. AAPS PharmSciTech.

[51] Liu F., Zhuang X., Yang C., Li Z., Xiong S., Zhang Z., Li J., Lu C., Zhang Z. (2014). Characterization of preclinical in vitro and in vivo ADME properties and prediction of human PK using a physiologically based pharmacokinetic model for YQA-14, a new dopamine D3 receptor antagonist candidate for treatment of drug addiction. Biopharm. Drug Dispos..

[52] Poulin P., Jones R.D.O., Jones H.M., Gibson C.R., Rowland M., Chien J.Y., Ring B.J., Adkison K.K., Ku M.S., He H. (2011). PHRMA CPCDC initiative on predictive models of human pharmacokinetics, Part 5: Prediction of plasma concentration-time profiles in human by using the physiologically-based pharmacokinetic modeling approach. J. Pharm. Sci..

[53] Williams H.D., Trevaskis N.L., Charman S.A., Shanker R.M., Charman W.N., Pouton C.W., Porter C.J.H. (2013). Strategies to address low drug solubility in discovery and development. Pharmacol. Rev..

